# Use-Exposure Relationships of Pesticides for Aquatic Risk Assessment

**DOI:** 10.1371/journal.pone.0018234

**Published:** 2011-04-01

**Authors:** Yuzhou Luo, Frank Spurlock, Xin Deng, Sheryl Gill, Kean Goh

**Affiliations:** Department of Pesticide Regulation, California Environmental Protection Agency, Sacramento, California, United States of America; Ghent University, Belgium

## Abstract

Field-scale environmental models have been widely used in aquatic exposure assessments of pesticides. Those models usually require a large set of input parameters and separate simulations for each pesticide in evaluation. In this study, a simple use-exposure relationship is developed based on regression analysis of stochastic simulation results generated from the Pesticide Root-Zone Model (PRZM). The developed mathematical relationship estimates edge-of-field peak concentrations of pesticides from aerobic soil metabolism half-life (AERO), organic carbon-normalized soil sorption coefficient (KOC), and application rate (RATE). In a case study of California crop scenarios, the relationships explained 90–95% of the variances in the peak concentrations of dissolved pesticides as predicted by PRZM simulations for a 30-year period. KOC was identified as the governing parameter in determining the relative magnitudes of pesticide exposures in a given crop scenario. The results of model application also indicated that the effects of chemical fate processes such as partitioning and degradation on pesticide exposure were similar among crop scenarios, while the cross-scenario variations were mainly associated with the landscape characteristics, such as organic carbon contents and curve numbers. With a minimum set of input data, the use-exposure relationships proposed in this study could be used in screening procedures for potential water quality impacts from the off-site movement of pesticides.

## Introduction

As part of the registration process, pesticides are evaluated for their potential to move off-site and impact non-target organisms. Surface runoff and tile flow are significant pathways for pesticides movement to surface waters. Monitoring-based surface water risk assessments of pesticides are usually conducted at the watershed scale using measured concentration data from river sites, especially at watershed outlets. For example, in-stream measurements of pesticides were assessed for U.S. watersheds with spatial scales across 14 orders of magnitude [1], [2]. However, water flow from non-application areas and non-agricultural headwaters may significantly dilute pesticide concentrations in the river. For example, in California's Central Valley, one of the most productive agricultural areas in the world, pesticide concentrations are substantially higher in small creeks dominated by irrigation return flows, as compared to main streams where the majority of flow originates in Sierra Nevada mountains [3], [4], [5], [6]. Because of the dilution effects, data in larger streams are associated with great spatial variability and thus not able to provide reliable and comparative information for pesticide management and mitigation. Therefore, assessments of aquatic risk now generally focus on smaller water bodies close to the edge-of-field.

Monitoring data are not always available and adequate for risk assessment, especially for pesticide products with new active ingredients. Environmental fate and transport models may be used to predict likely concentrations and associated risks of pesticides and to determine priorities for monitoring and regulatory assessments. Water quality modeling is a key component of pesticide management, as in the development of Best Management Practices (BMPs) and Total Maximum Daily Loads (TMDL). Compared to watershed-scale models, field-scale models better account for hydrologic processes within agricultural fields and have the capability to simulate agricultural management practices. Field-scale models, such as the Pesticide Root Zone Model (PRZM) and the Root Zone Water Quality Model (RZWQM) [7], provide dynamic simulation of pesticide fate and transport processes, from pesticide applications to edge-of-field discharge. However, these models usually require a large set of model input parameters for the full descriptions of landscape characteristics, climate conditions, and management practices [8]. Consequently parameterization and simulation using field-scale models could be complicated and time-consuming, particularly when batch simulations and post-data analysis are involved [9]. In addition, during the pesticide registration process, the required input data may be difficult to obtain, especially for new pesticides which have not been applied in field conditions. Therefore, there is a research need to develop simple mathematical relationships to determine the potential aquatic risks of pesticides based on a minimum set of input parameters. Such simple relationships may be used in a screening procedure to identify pesticides that require more refined studies. As an early modeling effort, U.S. Environmental Protection Agency (USEPA) developed the model for Generic Estimated Environmental Concentration (GENEEC) to mimic more sophisticated simulations of pesticide transport from crop field to a standard pond [10]. However, differences in climate, soil, topography or crop are not considered in estimating potential exposure, thus substantially limiting its applications to pesticide evaluation and registration.

This study develops “use-exposure relationships” in the form of linear regression equations that link pesticide application rate and physicochemical properties to a predicted exposure level (such as peak concentration) for specific environmental configurations. The relationship is developed from the results of more detailed field-scale model simulations, but use significantly fewer input parameters. Specific study objectives are [1] to identify governing parameters in pesticide fate and transport processes in canopy-soil system [2]; to establish empirical relationships between those parameters; and [3] to demonstrate the developed model with parameterizations in the crop scenarios of California. The approach enables a quick risk assessment based on limited input data, and yields accuracy comparable to the dynamic simulation of the selected field scale model. The parameterized use-exposure relationship provides useful information for decision making in pesticide registration and management.

## Materials and Methods

### PRZM Model and Aquatic Exposure Assessment

PRZM is a one-dimensional compartmental model developed by USEPA for predicting pesticide movement in unsaturated soils [11]. It is designed to evaluate the influence of climate, soil properties, and management practices on pesticide transport and transformation processes, e.g., surface runoff, plant uptake, leaching, erosion, and volatilization. PRZM generates daily pesticides fluxes in both dissolved and adsorbed forms at the edge of fields. The resulting fluxes are useful for further analyses, such as aquatic risk assessment [12], loading calculation [13], and water quality evaluation [14]. PRZM has undergone validation and testing to field-scale runoff and leaching studies [15], [16]. An enhanced version of PRZM is being used for surface water and groundwater exposure assessments in the European Union [17].

PRZM was selected in this study based on its ability to simulate relevant governing processes of pesticide transport and because of its use by regulatory agencies in their pesticide exposure assessments [10], [17]. USEPA has also developed crop scenarios to facilitate the application of PRZM in risk assessment [18]. Those scenarios specify the environmental configurations for typical crops in major agricultural regions of U.S., including weather conditions, landscape characteristics, crop growth parameters, and soil properties.

To assess pesticide risks to aquatic organisms, an exposure index (EI) was defined in this study as follows. First, the estimated environmental concentrations (EEC) of pesticide in surface runoff and soil erosion were predicted as daily time series by PRZM. For dissolved pesticides, the exposure index was then calculated as the peak concentration of 4-day moving averages in the 1-in-3 year return period. This definition is consistent with the current regulatory surface water criteria for two widespread pesticidal surface water contaminates chlorpyrifos and diazinon [19], [20].

### Simulation Design and Input Data

Stochastic PRZM simulations were conducted to develop crop-scenario-specific “use-exposure relationships”, i.e., empirical equations for predicting edge-of-field pesticide runoff concentrations. The simulations were based on crop scenarios developed by USEPA for pesticide risk assessment. A single annual pesticide application, repeated every year during 1961–1990, was simulated for a specific scenario. Annually repeated applications were utilized to incorporate the effects of climatic and hydrologic variations on pesticide off-site movement. In addition, the 30-year simulation also accounted for the accumulation of persistent pesticides. For those pesticides, residues from previous applications may remain in the soil and add to the newly applied chemicals in the next year. A random application date was assigned to a PRZM simulation and pesticide was applied on the assigned date for each year in that simulation. The random date was generated within the pesticide's application season depending on the actual use pattern of the pesticide, such as dormant-season application, in-season application, and pre-emergent application. On each day of application, pesticide was applied at a fixed rate (a “base rate” or BASE, kg/ha as the active ingredient), which was an arbitrary small application rate for stochastic PRZM runs. A linear relationship was assumed between pesticide application rate and pesticide loadings from the field. A small base rate was used to avoid high predicted concentrations that exceed the water solubility (SOL) of the pesticide during the simulation. In this study, the base rate was set as 0.1 kg/ha. Preliminary simulations showed that, with base rate of 0.1 kg/ha, EECs were always lower than the corresponding SOL in all PRZM runs. Predicted concentrations should be compared to the SOL when applying the developed use-exposure relationships with actual label rates.

The chemical properties of aerobic soil metabolism half-life (AERO), organic carbon-normalized soil adsorption coefficient (KOC), and SOL are the governing factors for pesticide runoff potential in both dissolved and adsorbed phases [21], [22]. KOC and SOL are direct input parameters in PRZM, and AERO is used in calculating the model inputs of decay rate constants [23]. In PRZM and most of other field-scale models, SOL is considered only as an upper limit on the dissolved concentration. In addition, significant association between the two properties of KOC and SOL has been reported in several previous studies. For example, linear correlation (p<0.001) was confirmed between log-transformed KOC and SOL [24]. Similar linear relationships were also used to estimate KOC from SOL [11], [25]. Therefore, only the independent chemical properties of AERO and KOC were selected in this study for stochastic PRZM simulations. The two selected parameters were also used by other studies for estimating pesticide runoff potentials [21], [26], [27], [28].

The probability distributions for AERO and KOC were derived from a database of physiochemical property and reaction half-life complied by Spurlock [24] for 172 pesticides. Spurlock [24] suggested that log normality is a reasonable assumption for AERO and KOC, and that the two properties are independent (p = 0.551). Maximum likelihood estimation (MLE) was applied to estimate the distribution parameters (Table 1). Latin Hypercube Sampling (LHS) was used to generate random input data of AERO and KOC within 95% of cumulative frequency of the corresponding log normal distribution as defined in Table 1. For each PRZM run, the exposure index from the single pesticide application at base rate, denoted as EI_BASE, was calculated from the predicted daily EECs. Finally, the general mathematical relationship between the EI_BASE and input chemical properties of AERO and KOC for the particular crop scenario was developed based on regression analysis. The built-in Monte Carlo simulation in PRZM does not report daily time series of edge-of-field pesticide concentrations. In addition, a deficiency has been reported for the built-in Monte Carlo module in PRZM [29]. Therefore, LHS algorithm was taken from our previous study [30]; and a batch program was developed for stochastic PRZM runs and post-data analysis.

**Table 1:** Parameters for the log-normal distribution of aerobic soil metabolism half-life (AERO) and organic carbon-normalized soil sorption coefficient (KOC).

Variable	μ	σ	E	SD
AERO	3.44	1.99	226.01	1613.14
KOC	6.51	2.52	1.61e4	3.82e5

Notes:

[1] the parameter estimation was based on the median fate properties derived from registration studies of 172 pesticides [24].

[2] μ and σ are the mean and standard deviation of the data's natural logarithm, respectively; E and SD are the mean and standard deviation of the data, respectively.

### Crop Scenarios in California

Crop scenarios developed by USEPA for California were used for simulations. Available scenarios include standard crop scenarios [18], crop scenarios developed for organophosphate pesticide cumulative risk assessment [31], and crop scenarios developed for effects determinations for California listed endangered and threatened species [32]. Combined, approximately 30 scenarios are available for California, some of which are associated with pesticide use patterns with high runoff potential. These include crops with flood or furrow irrigation, winter rain season application, and pre-emergent herbicide application. These scenarios were selected in this study to demonstrate the development of the pesticide use-exposure screening model. Results of a statewide survey of California irrigation methods [33] indicated that field crops and tomatoes are dominated by flood and furrow irrigation. Scenarios of almond and turf were selected to represent wet season application and pre-emergent herbicide application, respectively. Tables 2 and 3 summarize the selected scenarios for PRZM simulations for California. A non-California scenario (Florida tomatoes) was also included in this study to compare/contrast results with a wetter climate.

**Table 2:** Overview of selected California crop scenarios developed by USEPA.

Crop scenario	Represented use pattern	Soil (hydrologic group)	Weather station
Alfalfa (OP)	Pasture, gravity irrigation	Sacramento clay (D)	Fresno
Almond (STD)	Dormant application	Manteca fine sandy loam (C)	Sacramento
Cotton (STD)	Field crop, gravity irrigation	Twisselman Clay (C)	Fresno
Sugar beet (OP)	Field crop, gravity irrigation	Ryde clay loam (C)	Fresno
Tomato (STD)	Tomato, gravity irrigation	Stockton clay (D)	Fresno
Turf (RLF)	Pre-emergent application	CapaySilty Clay Loam (D)	San Francisco
Wheat (RLF)	Grain, gravity irrigation	San Joaquin Loam (D)	Fresno
Tomato_FL (STD)	Tomato scenario in Florida	Riviera Sand (C)	West Palm Beach

Data source: USEPA Tier 2 crop scenarios for PRZM/EXAMS Shell [18], [31], [32]. “STD” = Standard crop scenarios, “OP” = scenarios developed for the cumulative risk assessment of organophosphate pesticides, and “RLF” = scenarios developed for the effects determinations for the California red-legged frog and other California listed species. “Tomato_FL” denotes the standard USEAP crop scenario for tomato in Florida, provided as an example of the crop scenarios in other states.

**Table 3:** Landscape characteristics and soil properties of selected California crop scenarios.

Crop scenario	CN	USLE K/LS/P	USLE C	OC1
Alfalfa	90/88/89	0.20/0.30/1.0	0.051–0.217	1.77%
Almond	84/79/84	0.28/0.30/1.0	0.034–0.221	0.81%
Cotton	89/86/89	0.21/0.37/1.0	0.054–0.412	0.29%
Sugar beet	89/86/89	0.28/0.30/1.0	0.015–0.769	3.48%
Tomato	91/87/91	0.24/0.13/1.0	0.035–0.255	0.95%
Turf	80/80/80	0.37/1.80/0.5	0.001	35.6%
Wheat	92/89/90	0.37/0.79/1.0	0.027–0.604	0.44%
Tomato_FL	91/87/91	0.03/0.20/1.0	0.177–0.938	1.16%

Parameters:

CN = Runoff curve numbers of antecedent moisture condition II for fallow, cropping, and residue, respectively;

USLE K = soil erodibility for the universal soil loss equation (USLE);

USLE LS = topographic factor for the USLE;

USLE P = practice factor for the USLE;

USLE C = cover management factor for the USLE;

OC1 = Organic carbon content in the surface soil.

The crop scenarios specify the weather conditions, soil properties, and crop growth parameters used in the PRZM simulations. Other model inputs used in this study, including chemical property and pesticide application, are summarized in Table 4. To provide conservative estimation of pesticide residues at the edge of field, pesticides were assumed to be incorporated into the soil at application and all mass loss fluxes by interception, volatilization, and decay on the plant canopy were set as zero.

**Table 4:** Chemical property and pesticide application in PRZM simulations.

Variable	Description	Values/notes
APPDAY	Application date	Random numbers (uniform) in the application season
APPEFF	Application efficiency	0.99 (ground application) [2]
CAM	Pesticide application method	4 (soil incorporation)
DAIR	Diffusivity in air (cm2/day)	4300 [1]
DEPI	Incorporation depth (cm)	4 [2]
DRFT	Drift fraction	0.01 [2]
DSRATE	Adsorbed phase decay rate (1/d)	= ln2/AERO, LHS sampling
DWRATE	Dissolved phase decay rate (1/d)	= DSRATE [2]
ENPY	Enthalpy of vaporization (kcal/mol)	20 [1]
FEXTRC	Washoff extraction (1/cm)	0.5 [1]
HENRYK	Henry's law constant (g/aq, dimensionless)	0 [3]
IPSCND	Disposition of foliar pesticide after harvest	1 (surface applied) [3]
PLDKRT	Decay rate on foliage (1/d)	0 [2]
PLVKRT	Volatilization rate on foliage (1/d)	0 [2]
KOC	Organic carbon-normalized soil adsorption coefficient (L/kg[OC])	LHS sampling
TAPP	Application rate (kg/ha)	0.1 (base application rate used in this study)
UPTKF	Pesticide uptake	0 [2]

Notes:

[1]Suggested value in the PRZM manual [11].

[2]USEPA-suggested model input parameter value [23].

[3]Assumptions made for conservative evaluation of pesticide exposure.

## Results and Discussion

### Use-Exposure Relationship for a Single Pesticide Application

**Figure 1:**
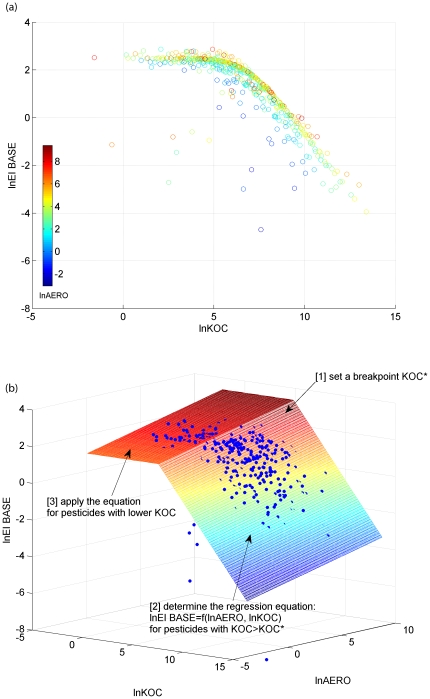
Use-exposure relationship for dissolved pesticides (EI_BASE in µg/L): (a) example results of Monte Carlo simulation and (b) conceptual model.

The response of EI_BASE to random values of AERO and KOC was evaluated by stochastic PRZM simulation. For each crop scenario, the predicted dissolved or adsorbed EI_BASE were paired with corresponding inputs of AERO and KOC for further regression analysis. The logarithmic transformation was also applied to EI_BASE according to preliminary analyses on pesticide concentrations detected in surface water of California [34]. Finally, an N×3 matrix of (lnAERO, lnKOC, lnEI_BASE), with N denoting the number of stochastic PRZM runs, was generated from Monte Carlo simulation. Demonstrated in Figure 1a is an example plot of the matrix for dissolved pesticides for the standard crop scenario for cotton in California. Significant correlations were identified between dissolved EI_BASE vs. AERO and KOC, especially for pesticides with KOC higher than a certain value (e.g., about 5 for lnKOC as shown in Figure 1a). This correlation reflected the effects of degradation and partitioning of pesticides on the peak concentration predicted at the field edge. For pesticides with lower KOC, EI_BASE was generally invariant with KOC. With low KOC values, pesticides are mainly present in dissolved phase, thus the change in KOC do not have a great effect on the phase partitioning.

Stochastic PRZM simulations for other crop scenarios revealed similar relationships among the predicted EI_BASE and input parameters, i.e., the general linear relationship between EI_BASE and AERO and KOC, and the presence of an approximate lnKOC cutoff, below which EI_BASE is independent of KOC. Therefore, a conceptual model was developed for the use-relationships from a single pesticide application based on that general data structure (Figure 1b). First, a breakpoint for KOC (KOC*) was determined from the trend of dissolved EI_BASE with KOC for the given crop scenario. Multivariate linear regression with logarithmic transformations was conducted between EI_BASE vs. AERO and KOC on the data points with KOC>KOC*. A similar relationship was applied to pesticides with KOC≤KOC* by substituting KOC with KOC*, in order to provide conservative estimation of EI_BASE for those pesticides.

The general use-exposure relationship is:
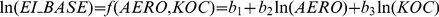
(1)And the relationship for dissolved pesticides is:
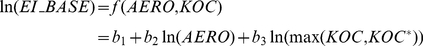
(2)where b_1_, b_2_, and b_3_ are coefficients derived by regression. KOC* was determined by maximizing the coefficient of determination (R^2^) in the regression analysis. Based on the linear assumption between pesticide application and exposure, the dissolved exposure index (EI, µg/L) from pesticide applications at the actual rate (RATE, kg/ha) was expressed as,
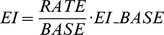
(3)


### Derived Parameters for Dissolved Pesticides

For each selected scenario, 5 000 stochastic simulations of PRZM (N = 5 000) were conducted for the 30-year period of 1961–1990. Regression coefficients and other statistics for the use-exposure relationship for dissolved pesticides are summarized in Table 5.

**Table 5:** Use-exposure relationships for dissolved pesticides in selected California crop scenarios.

Scenarios	Coefficients			R^2^	lnKOC*
	b1	b2	b3		
Alfalfa	5.2156	0.1907	−0.8288	0.9494	3.5
Almond	4.8131	0.1869	−0.7467	0.9335	4.5
Cotton	6.3173	0.1467	−0.7662	0.9102	5.5
Sugar beet	4.9105	0.2412	−0.8377	0.9193	3.0
Tomato	5.9979	0.1785	−0.7844	0.8970	4.0
Turf	3.3647	0.2821	−0.8248	0.9546	0.5
Wheat	6.0764	0.1853	−0.7954	0.9487	5.0
Tomato_FL	4.9362	0.2531	−0.8063	0.9422	4.0

Note: “Tomato_FL” denotes the standard USEAP crop scenario for tomato in Florida, which is provided as an example of the crop scenarios in other states.

Values of lnKOC* varied with different scenarios, ranging from 0.5 to 5.5. With higher organic carbon content in surface soil (OC1), such as for the California turf scenario (35.6%), lower KOC* values were observed relative to other scenarios with smaller OC1 ranging from 0.44% to 3.48% (Table 3). However, the product of KOC* and OC1, equivalent to the corresponding limiting distribution coefficient (KD*), was approximately constant among the scenarios, ranging from 0.5 to 0.7. Since the distribution coefficients indicate pesticide mobility in the soil, the KD* value determined from the PRZM simulations was considered as the critical KD value below which the transport process of dissolved pesticides with surface runoff was insensitive to their KOC values.

The empirical AERO-KOC based use-exposure relationships explained 90–95% of the variances on the predicated EI_BASE of dissolved pesticides. The predictive ability of the relationships was mainly attributable to lnKOC, which solely explained 85–90% of the total variances in lnEI_BASE, while AERO had only a limited contribution. Generally, the relative magnitudes of dissolved EI_BASE for pesticides with large KOC were mainly related to the regression coefficients of KOC (b3), while those for pesticides with lower KOC were determined by the intercepts (b1). This reflected the competition between phase partitioning and water runoff extraction on the pesticide yields from the applied field. Relatively higher regression coefficients for AERO (b2) were observed for scenarios with higher OC1 such as sugar beet (OC1 = 3.48%) and turf (OC1 = 35.6%) (Table 3). With elevated OC contents, pesticides are less mobile in the soil and could be accumulated for a longer period. Previous studies indicated that pesticide half-life in the soil is the key parameter in determining the total amount of pesticide residues discharged from fields [21], [22], [35]. However, the measure of exposure in risk characterization is estimated from peak concentrations, which are usually observed shortly after pesticide applications once surface runoff induced by precipitation or irrigation is available. Thus, soil metabolism might have only moderate effects on pesticide exposure at the edge of field as measured by peak concentrations.

The regression coefficients for AERO and KOC did not vary much over scenarios. For instance, the maximum regression coefficient for KOC was −0.7467 (almonds), while the minimum value was −0.8377 (sugar beet). The regression coefficients for AERO ranged from 0.1467 (cotton) to 0.2821 (turf) (Table 5). The use-exposure relationship derived for crops in other states, taking tomato in Florida as an example in this study, also showed similar regression coefficients as in California crops. This suggested that, for a specific pesticide, the difference of predicted EI_BASE over scenarios were mainly determined by the intercepts of b1 in Eq. (1). In another words, the effects of chemical fate processes such as partitioning and degradation on pesticide exposure were similar among scenarios, while the spatial variability was related to environmental parameters including climate condition, soil property, and landscape characteristics.

While the California scenarios are developed for areas with similar climate, they are associated with substantial variability in soil type and hydrologic group (Table 3). The intercept in the regression equation for the use-exposure relationship (b1 in Table 5) was significantly correlated to curve numbers for residue surface soil condition (with a p-value, p = 0.008), and moderately correlated to curve numbers for cropping surface condition (p = 0.063). In most of the crop scenarios, curve numbers for residue surface condition were implemented for winter months, or the rainfall season in California. In the use-exposure relationship, the intercept was associated with water runoff generation since the chemical fate processes such as partitioning and degradation were represented by the chemical properties. Therefore, the significant correlation between b1 and curve number for residue surface soil condition indicated that peak concentrations of pesticide at the field edges were most likely observed during the rainfall season in California. The dependence of pesticide runoff potential on curve number in the PRZM simulation has been reported in previous studies [22], [36], [37]. Findings in this study confirmed the effects of curve number on the predicted pesticide concentrations and loadings from the crop fields.

### Derived Parameters for Sediment-Bound Pesticides

At present there are no surface water quality criteria at either federal or state level for sediment-bound pesticides. Water quality assessments for pesticides in sediment, such as those for Clean Water Act Section 303(d) listing [38], are based on 10-day *Hyalella azteca* sediment toxicity tests [39]. To mimic the sediment toxicity tests, 10-day averages were calculated as adsorbed exposure index from PRZM-predicted daily concentrations of pesticide associated with soil erosion. The same frequency as for dissolved pesticide, i.e., once every three years return period, was used in the development of use-exposure relationship for adsorbed pesticides. Median lethal concentration (LC50) values for sediment toxicity are usually reported on an OC-normalized basis. For example, *Hyalella azteca* 10-day LC50 values for pyrethroids are typically reported at 1% OC, as compiled by Domagalski et al. [40]. To match the toxicity data, PRZM-predicted concentrations of pesticide in eroded sediment were normalized by OC1 defined in each scenario (Table 3).

**Figure 2:**
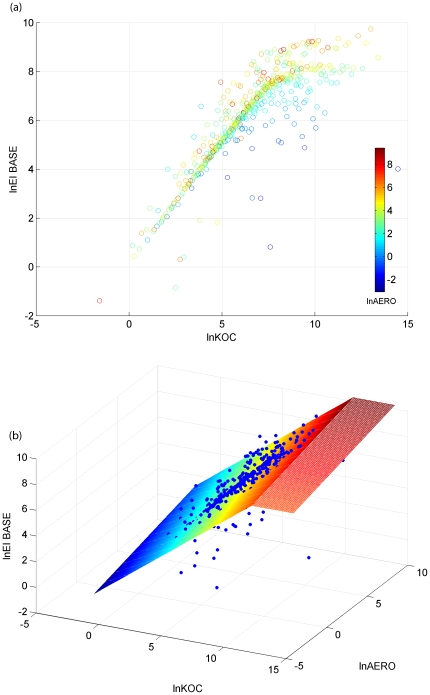
Use-exposure relationshio for adsorbed pesticides (EI_BASE in ng/g): (a) example results of Monte Carlo simulation and (b) conceptual model.

For pesticide associated with eroded soil, Figure 2 shows the results of stochastic PRZM runs based on the USEPA standard scenario for cotton in California. There was a general increasing trend of EI_BASE with increases of KOC and AERO, especially for pesticides with KOC lower than a certain value. For pesticides with extremely high values, such as those with lnKOC larger than about 11 as shown in Figure 2a, the predicted EI_BASE for adsorbed pesticides were associated with high uncertainty and might not be significantly correlated with their KOC values. For these pesticides, the majority of the residues have been partitioned into the particulate phase. Based on the similar equations for dissolved pesticides, the following use-exposure relationship was developed for adsorbed pesticides,
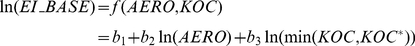
(4)where EI_BASE (ng/g) is the predicted exposure index associated with eroded soil from a single pesticide application at BASE rate of 0.1 kg/ha, b's are regression coefficients, and KOC* is a threshold value for KOC above which the EI_BASE was assumed to be independent the pesticide's KOC.

Table 6 shows the parameters of the use-exposure relationships for adsorbed pesticides under selected California scenarios. The R^2^ values ranged from 60–85%, substantially lower than those for dissolved pesticides (Table 5). Similar to the equations for dissolved pesticides, the majority of the variance in the use-exposure relationships for adsorbed pesticides wasexplained by KOC. Although the KOC* varied greatly, the KD* values as the product of KOC* and OC1 for the modeled scenarios were generally invariant, ranging from 160–240. However, the regression coefficients varied greatly among scenarios. Observed uncertainty in predicted exposure of adsorbed pesticides might be related to soil erosion processes. PRZM simulates soil erosion based on themodified universal soil loss equation (MUSLE) with input parameters. These parameters are usually associated with seasonality and variability in soil properties and management practices (Table 3). With only input parameters of AERO and KOC, therefore, the proposed use-exposure relationship was inadequate for capturing the variability in soil erosion processes.

**Table 6:** Use-exposure relationships for adsorbed pesticides in selected California crop scenarios.

Scenarios	Coefficients			R^2^	ln(KOC*)
	b1	b2	b3		
Alfalfa	1.7756	0.3140	0.4936	0.6896	9.5
Almond	0.1179	0.2116	0.6937	0.7955	10.0
Cotton	0.9213	0.1890	0.7221	0.8466	11.0
Sugar beet	2.7386	0.3254	0.5118	0.6409	8.5
Tomato	3.2070	0.1912	0.6062	0.7770	10.0
Turf	2.7715	0.2832	0.4486	0.6106	6.5
Wheat	1.0782	0.3233	0.5848	0.7210	10.5
Tomato_FL	1.7065	0.4105	0.4809	0.7607	10.0

### Use-Exposure Relationship for Multiple Applications

The use-exposure relationships in Eqs. (1)through(4) provide estimates for the exposure index from a single pesticide application. For multiple applications, the exposure could be conservatively estimated from the maximum season application rate. To refined the estimation, especially for pesticides with short field dissipation half-lives (FD), equivalent application rate at any given time, RATE_eq(t), is calculated as the total pesticide amount in the soil available for runoff and soil erosion processes. RATE_eq includes contributions from both applied pesticide on the given day and residues from previous applications, and could be calculated in the form of a convolution,
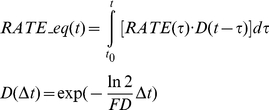
(5)where t0 is the first day of the application season, RATE(t) is the application rate at day t, and D(Δt) is the total fractional decay during Δt. The calculation of RATE_eq accounted for only the pesticide loss by degradation. Losses from surface runoff, soil erosion, and leaching were ignored to provide a conservative estimation of the amount of pesticide remaining in the soil.

**Figure 3:**
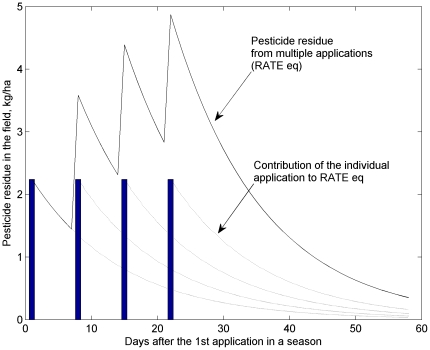
Equivalent application rate from multiple pesticide applications, illustrated with recommended application rates and intervals of carbaryl for tomatoes[**32**]. Bars represent four applications at 2.24 kg/ha and 7-day intervals.

Figure 3 presents a schematic of the RATE_eq calculation from multiple applications of carbaryl for tomatoes (maximum single application rate = 2.24 kg/ha, application interval = 7 day, and maximum number of applications = 4) [32]. The exposure index from multiple pesticide applications could be estimated by substituting RATE in Eq. (3) with the maximum value of RATE_eq during a year or a cropping season. For single pesticide application rate and application interval (INTERVAL, day) as fixed values, as usually documented in pesticide labels, the maximum RATE_eq could be directly calculated as,
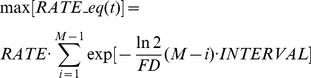
(6)where M is the maximum number of applications.

### Summary and Conclusions

Use-exposure relationships were developed as an alternative approach to field-scale modeling for pesticide runoff and associated aquatic risks. The relationships require a minimum set of input parameters to estimate exposure, which is defined herein as peak pesticide concentrations at the edge of field. The selected input parameters, half-life in the soil, adsorption coefficient, and recommended application rates, are generally available during pesticide registration. Thus the proposed approach is appropriate for quickly screening pesticide products for their potential adverse effects on the environment and human health. While the PRZM model was chosen to parameterize the weighting factors of the selected parameters for this study, the approach could be applied with other field-scale models.

The development of use-exposure relationships was demonstrated using crop scenarios developed by the USEPA for California. The relationships explained 90–95% of variations in the exposure index of dissolved pesticides as predicted by PRZM modeling. Regression coefficients for AERO and KOC for the simulated scenarios varied only in small ranges, suggesting that the effects of chemical property-related fate processes such as partitioning and degradation on the predicted exposure index were similar among scenarios. KOC was the governing factor in predicting pesticide exposures for all scenarios. Since aquatic risk analysis is mainly focused on the peak concentrations of pesticides, and these concentrations are usually observed shortly after pesticide applications, the half-life in the soil had limited influence on the exposure index defined in this study. The results of this study suggested that the selection of evaluation approaches for pesticide exposure could be dependent on the purpose of regulatory assessment and management planning. For instance, total pesticide loadings from agricultural fields might be very sensitive to chemical persistence, while the peak concentrations of a pesticide are mainly related to its mobility. For a particular pesticide, the spatial variability on its exposure indices across various scenarios is associated with landscape characteristics, such as OC and CN values. In addition, significant correlation was observed between intercept constants (b1) in the use-exposure relationships and the curve numbers for residue in the crop scenarios. Those findings indicated the possibility in developing generic equations of use-exposure relationships.

Methods to extend the capability of use-exposure relationship modeling are provided, including applications for assessing sediment-bound pesticides and provisions for multiple pesticide applications within a growing season. Since the use-exposure relationships were parameterized from regression analysis on the simulation results of existing field-scale models, the accuracy of risk assessment based on those relationships is associated with the model itself and the configuration of crop scenarios. Future work issuggested to implement the developed equations in real field conditions and to compare the predictions with measured pesticide data. An evaluation of measured data with corresponding field conditions would generate instructive information for developing crop scenarios for pesticide exposure assessment and risk characterization.

## References

[1] Capel PD, Larson SJ, Winterstein TA (2001). The behaviour of 39 pesticides in surface waters as a function of scale.. Hydrologic Processes.

[2] Capel PD, Larson SJ (2001). Effect of scale on the behavior of atrazine in surface waters.. Environmental Science & Technology.

[3] Domagalski JL, Munday C (2003). Evaluation of diazinon and chlorpyrifos concentrations and loads, and other pesticide concentrations, at selected sites in the San Joaquin Valley, California, April to August, 2001..

[4] Kratzer CR, Zamora C, Knifong DL (2002). Diazinon and chlorpyrifos loads in the San Joaquin River Basin, California, January and February 2000..

[5] Dubrovsky NM, Kratzer CR, Brown LR, Gronberg JM, Burow KR (1998). Water quality in the San Joaquin-Tulare Basins, California, 1992–95..

[6] Ross LJ, Stein R, Hsu J, White J, Hefner K (1999). Distribution and mass loading of insecticides in the San Joaquin River, California.

[7] Ahuja LR, Rojas KW, Hanson JD, Shaffer MJ, Ma L (1999). Root zone water quality model – Modeling management effects on water quality and crop production.

[8] USGS (2005). Evaluation of unsaturated-zone solute-transport models for studies of agricultural chemicals, Open-File Report 2005-1196 (http://pubs.usgs.gov/of/2005/1196/ofr20051196.pdf, accessed 02/2011).

[9] Nolan BT, Bayless ER, Green CT, Garg S, Voss FD (2005). Evaluation of Unsaturated-Zone Solute-Transport Models for Studies of Agricultural Chemicals, Open-File Report 2005-1196.

[10] USEPA (2010). Water exposure models used by the Office of Pesticide Programs (http://www.epa.gov/oppefed1/models/water/models4.htm, accessed 10/2010).

[11] USEPA (2006). PRZM-3, a model for predicting pesticide and nitrogen fate in the crop root and unsaturated soil zones: users manual for release 3.12.2.

[12] Luo Y, Zhang M (2009). A geo-referenced modeling environment for ecosystem risk assessment: organophosphate pesticides in an agriculturally dominated watershed.. Journal of Environmental Quality.

[13] Dasgupta S, Cheplick JM, Denton DL, Troyan JJ, Wiliams WM, Gan J, Spurlock F, Hendley P (2008). Predicted runoff loads of permethrin to the Sacramento River and its tributaries. Synthetic Pyrethroids, Occurrence and Behavior in Aquatic Environments.

[14] Cryer SA, Fouch MA, Peacock AL, Havens PL (2001). Characterizing agrochemical patterns and effective BMPs for surface waters using mechanistic modeling and GIS.. Environmental Modeling and Assessment.

[15] Jones RL, Russell MH (2001). Final Report of FIFRA Environmental Model Validation Task Force (http://femvtf.com/femvtf/Files/FEMVTFbody.pdf, accessed 10/2010).

[16] Singh P, Jones RL (2002). Comparison of pesticide root zone model 3.12: Runoff predictions with field data.. Environmental Toxicology and Chemistry.

[17] FOCUS (2001). FOCUS surface water scenarios in the EU evaluation process under 91/414/EEC (http://focus.jrc.ec.europa.eu/, accessed 10/2010).. http://focus.jrc.ec.europa.eu/.

[18] USEPA (2008). USEPA Tier 2 crop scenarios for PRZM/EXAMS Shell (http://www.epa.gov/oppefed1/models/water/index.htm, accessed 09/2010).

[19] Siepmann S, Finlayson B (2000). Water quality criteria for diazinon and chlorpyrifos. Administrative Report 00-3.

[20] USEPA (2005). Aquatic Life Ambient Water Quality Criteria, Diazinon (EPA-822-R-05-006)..

[21] Goss EW (1992). Screening procedure for soils and pesticides for potential water quality impacts.. Weed Technology.

[22] Luo Y, Zhang M (2010). Spatially distributed pesticide exposure assessment in the Central Valley, California, USA.. Environmental Pollution.

[23] USEPA (2002). Guidance for selecting input parameters in modeling the environmental fate and transport of pesticides, version II.

[24] Spurlock F (2008). Distribution and variance/covariance structure of pesticide environmental fate data.. Environmental Toxicology and Chemistry.

[25] Chapra SC (1997). Surface water-quality modeling..

[26] Chen W, Hertl P, Chen S, Tierney D (2002). A pesticide surface water mobility index and its relationship with concentrations in agricultural drainage watersheds.. Environmental Toxicology and Chemistry.

[27] Kellogg RL, Nehring R, Grube A, Goss DW, Plotkin S (2000). Environmental Indicators of Pesticide Leaching and Runoff from Farm Fields (http://www.nrcs.usda.gov/technical/NRI/pubs/eip_pap.html, accessed 11/2010).

[28] Larson SJ, Crawford CG, Gilliom RJ (2004). Development and application of Watershed Regressions for Pesticides (WARP) for estimating atrazine concentration distributions in streams, Water-Resources Investigations Report 03-4047.

[29] USEPA (2010). Pesticide Root Zone Model (PRZM) release notes (http://www.epa.gov/ceampubl/gwater/przm3/prz3reln.html, accessed 10/2010).

[30] Luo Y, Yang X (2007). A multimedia environmental model of chemical distribution: fate, transport, and uncertainty analysis.. Chemosphere.

[31] USEPA (2006). Organophosphate pesticides: revised cumulative risk assessment (http://www.epa.gov/pesticides/cumulative/rra-op/, accessed 10/2010).

[32] USEPA (2010). Effects Determinations for the California Red-legged Frog and other California Listed Species (http://www.epa.gov/espp/litstatus/effects/redleg-frog/index.html, assessed 02/2011).

[33] CDWR (2002). 2001 Statewide Irrigation Methods Survey (http://www.water.ca.gov/landwateruse/, accessed 09/2010).

[34] CEPA (2010). Surface Water Database.

[35] Villeneuve J-P, Lafrance P, Banton O, Frechette P, Robert C (1988). A sensitivity analysis of adsorption and degradation parameters in the modeling of pesticide transport in soils.. Journal of Contaminant Hydrology.

[36] Wolt J, Singh P, Cryer S, Lin J (2002). Sensitivity analysis for validating expert opinion as to ideal data set criteria for transport modeling.. Environmental Toxicology and Chemistry.

[37] Don DF, Havens PL, Blau GE, Tillotson PM (1992). The Role of Sensitivity Analysis in Groundwater Risk Modeling for Pesticides.. Weed Technology.

[38] CEPA (2010). 2010 Integrated Report (Clean Water Act Section 303(d) List/305(b) Report (http://www.waterboards.ca.gov/water_issues/programs/tmdl/integrated2010.shtml, accessed 11/2010).

[39] USEPA (1999).

[40] Domagalski JL, Weston DP, Zhang M, Hladik M (2010). Pyrethroid insecticide concentrations and toxicity in streambed sediments and loads in surface waters of the San Joaquin Valley, California, USA.. Environmental Toxicology and Chemistry.

